# Alpha-Tomatine Attenuation of *In Vivo* Growth of Subcutaneous and Orthotopic Xenograft Tumors of Human Prostate Carcinoma PC-3 Cells Is Accompanied by Inactivation of Nuclear Factor-Kappa B Signaling

**DOI:** 10.1371/journal.pone.0057708

**Published:** 2013-02-21

**Authors:** Sui-Ting Lee, Pooi-Fong Wong, Hui He, John David Hooper, Mohd Rais Mustafa

**Affiliations:** 1 Department of Pharmacology, Faculty of Medicine, University of Malaya, Lembah Pantai, Kuala Lumpur, Malaysia; 2 Mater Medical Research Institute, South Brisbane, Queensland, Australia; University of Illinois at Chicago, United States Of America

## Abstract

**Background:**

Nuclear factor-kappa B (NF-κB) plays a role in prostate cancer and agents that suppress its activation may inhibit development or progression of this malignancy. Alpha (α)-tomatine is the major saponin present in tomato (*Lycopersicon esculentum*) and we have previously reported that it suppresses tumor necrosis factor-alpha (TNF-α)-induced nuclear translocation of nuclear factor-kappa B (NF-κB) in androgen-independent prostate cancer PC-3 cells and also potently induces apoptosis of these cells. However, the precise mechanism by which α-tomatine suppresses NF-κB nuclear translocation is yet to be elucidated and the anti-tumor activity of this agent *in vivo* has not been examined.

**Methodology/ Principal Findings:**

In the present study we show that suppression of NF-κB activation by α-tomatine occurs through inhibition of I kappa B alpha (IκBα) kinase activity, leading to sequential suppression of IκBα phosphorylation, IκBα degradation, NF-κB/p65 phosphorylation, and NF-κB p50/p65 nuclear translocation. Consistent with its ability to induce apoptosis, α-tomatine reduced TNF-α induced activation of the pro-survival mediator Akt and its inhibition of NF-κB activation was accompanied by significant reduction in the expression of NF-κB-dependent anti-apoptotic (c-IAP1, c-IAP2, Bcl-2, Bcl-xL, XIAP and survivin) proteins. We also evaluated the antitumor activity of α-tomatine against PC-3 cell tumors grown subcutaneously and orthotopically in mice. Our data indicate that intraperitoneal administration of α-tomatine significantly attenuates the growth of PC-3 cell tumors grown at both sites. Analysis of tumor material indicates that the tumor suppressing effects of α-tomatine were accompanied by increased apoptosis and lower proliferation of tumor cells as well as reduced nuclear translocation of the p50 and p65 components of NF-κB.

**Conclusion/ Significance:**

Our study provides first evidence for *in vivo* antitumor efficacy of α-tomatine against the human androgen-independent prostate cancer. The potential usefulness of α-tomatine in prostate cancer prevention and therapy requires further investigation.

## Introduction

Prostate cancer is the second most frequently diagnosed cancer and the sixth leading cause of cancer death in men worldwide [1]. As progression of this malignancy is dependent on the androgen receptor, therapies that target activating ligands (the hormones testosterone and dihydrotestosterone) produce response rates in patients of up to 95% [2]. Unfortunately, nearly all prostate cancer patients develop hormone-refractory prostate cancer (HRPC) [2]. For these patients curative treatments are not available and docetaxel-based chemotherapy provides palliation with response rates of approximately 50% and median survival of 18 to 20 months with survival benefit of about 2 months [3]. For patients with HRPC, low toxicity molecular targeting strategies are needed.

Accumulating evidence suggests that the transcription factor nuclear factor-kappa B (NF-κB) plays a pivotal role in prostate cancer growth, survival, angiogenesis and metastatic progression [4], [5], [6], [7], [8]. NF-κB consists of a p50/p65 heterodimer, that is masked by the inhibitor of NF-κB, I kappa B alpha (IκBα) that causes its retention in the cytoplasm under resting condition. Various stimuli, including tumor necrosis-alpha (TNF-α), phorbol ester and lipopolysaccharides (LPS), result in IκBα kinase activation, which mediates IκBα phosphorylation at Ser32 and Ser36 followed by its ubiquitination and proteasome-mediated degradation. This releases the NF-κB p50/p65 heterodimer, which then translocates to the nucleus, where it binds to consensus sequence motifs to induce gene transcription. It has been demonstrated that NF-κB is constitutively activated in androgen-insensitive prostate carcinoma cells, and overexpression of NF-κB p65 protein was found in the nuclear fraction of prostate cancer clinical specimens [5], [9], suggesting a role for NF-κB in prostate cancer progression. Consistently, it has been report that aberrant IKK activation leads to the constitutive activation of the NF-κB survival pathway in androgen-independent prostate cancer cells [10]. In addition, activation and localization of NF-κB represent independent risk factors for disease recurrence after radical prostatectomy [9], [11]. Hence, effective inhibition of NF-κB could be a promising strategy for treatment of prostate cancer and prevention of relapse.

Alpha (α)-tomatine is the major saponin in tomato (*Lycopersicon esculentum*). Previous studies have reported its immunopotentiating [12] and *in vitro* anti-cancer activities [13], [14], [15], [16]. It also has protective effects against dibenzo[a,l]pyrene (DBP)-induced liver and stomach tumors in rainbow trout without causing significant changes in total weight, liver weight, tissue morphology and mortality [17]. Thus far, the mechanism by which α-tomatine mediates its anti-prostate cancer effect is not well understood. Our previous study reported the pro-apoptotic effect of α-tomatine against androgen-independent human prostatic adenocarcinoma PC-3 cells through the inhibition of TNF-α-induced NF-κB nuclear translocation [18]. In the present study, the mechanism of the inhibition of α-tomatine on NF-κB signaling pathway is further characterized. For the first time, this study demonstrates the potent anti-tumor activity of α-tomatine against human androgen-independent prostate cancer *in vivo*.

## Materials and Methods

### Ethics statement

Experiments with mice were performed in accordance with the protocol approved by the University of Queensland Animal Ethics Committee (AEC Approval Number: MMRI/210/10).

### Materials

α-tomatine was purchased from Tokyo Chemical Industry (Tokyo, Japan). Dimethyl sulfoxide (DMSO), TNF-α, fetal bovine serum (FBS), 3,3,5,5 tetramethylbenzidine (TMB), Calpain Inhibitor I, ALLN (N-acetyl-leucyl-leucyl-norleucinal) and anti-human beta (β)-actin antibody were purchased from Sigma Aldrich (St. Louis, MO). Penicillin/streptomycin, Roswell Park Memorial Institute (RPMI-1640) media and 0.4% trypan blue solution were purchased from Invitrogen (Carlsbad, CA). Protein A/G plus agarose beads, Akt inhibitor VIII, and antibodies against p65, p50, IκBα, Akt, IKKα, and IKKβ were obtained from Santa Cruz Biotechnology, CA. The glutathione *S*-transferase-IκBα (GST-IκBα) fusion protein and polyvinylidene fluoride (PVDF) membrane were purchased from Millipore (Bedford, MA). Kinase buffer, antibodies against phospho-specific IκBα (Ser32/36), phosphor-specific p65 (Ser536), phosphor-specific Akt (Ser473), B cell leukaemia-2 (Bcl-2), B cell leukaemia-x long (Bcl-xL), cellular inhibitor of apoptosis 1 (c-IAP1), cellular inhibitor of apoptosis 2 (c-IAP2), survivin, X-linked inhibitor of apoptosis (XIAP), histone H3 and cleaved-Poly (ADP-ribose) polymerase (PARP) antibodies were purchased from Cell Signaling (Beverly, MA). Antibodies against Ki-67 and proliferating cell nuclear antigen (PCNA) were purchased from BD Biosciences (San Diego, CA).

### Cell line

The prostate cancer PC-3 cell line was purchased from the American Type Culture Collection (Manassas, VA). Luciferase-expressing prostate cancer PC-3 cell line was a kind gift of Dr. Patrick Ming Tat Ling, Queensland University of Technology, Australia [19], [20]. Both PC-3 and luciferase-expressing PC-3 cells were cultured in RPMI-1640 supplemented with 10% FBS, 100 U/mL penicillin and 100 µg/mL streptomycin. Cells were cultured at 37°C in a 5% CO_2_ humidified incubator.

### Cell treatment and fractionation

For the *in vitro* assays, PC-3 cells at 70-80% confluency were treated with α-tomatine (2 µM) for 30 minutes, and then exposed to10 ng/ml TNF-α for various time periods. Akt inhibitor VIII (10 µM) which inhibits activation of Akt as evidenced by reduced phosphorylation of this kinase at Thr308 and Ser473 [21] was used as inhibitor control for studying the effect of α-tomatine on Akt activation as described previously [22]. Both nuclear and cytoplasmic fractions of treated and vehicle control cells were isolated using a nuclear extraction kit (Cayman Chemical, Ann Arbour, MI) according to the manufacturer’s instructions. Briefly, cells were harvested using a cell scrapper then pelleted by centrifugation at 4°C before two washes with ice-cold PBS supplemented with phosphatase inhibitor solution at 4°C. Pelleted cells were swollen for 15 minutes in ice-cold hypotonic buffer supplemented with complete protease and phosphatase inhibitors. 10 % Nonidet P-40 assay reagent was then added and cytosolic fractions were collected by brief centrifugation. Pellets were resuspended in ice-cold complete nuclear extraction buffer then vortexed on ice for 30 seconds at highest setting. These cell pelleting and vortexing steps were repeated for a total of 6 cycles. The final pellet was resuspended then centrifuged at 14,000 x g for 10 minutes at 4°C. The supernatants containing nuclear fractions were collected.

### Cell viability analysis

Cell viability was examined using a trypan blue exclusion assay as described previously [23]. Briefly, cells in control and treated groups were harvested, stained with 0.4% trypan blue solution and total viable cells were counted using hemacytometer. The proportion of viable cells was calculated by dividing the number of viable test cells by the number of viable control cells at the end of each experimental treatment.

### IκBα kinase assay

The effect of α-tomatine on TNF-α-induced IKK activation was analyzed as described previously [24]. Briefly, PC-3 cells were preincubated with either 2 µM α-tomatine or 0.1% DMSO (vehicle) for 30 minutes, and then treated with 10 ng/ml TNF-α for the indicated times. IKK complex was immunoprecipitated from whole cell extracts using antibodies against IKKα and IKKβ. Protein A/G plus agarose beads were added and incubated at 4°C for overnight. The beads were washed with lysis buffer and resuspended in a kinase buffer before GST-IκBα as IKK substrate was added. After incubation at 30°C for 30 minutes, the reaction was terminated by addition of Laemmli’s loading buffer and heated at 100°C for 5 minutes. Western blot analysis was performed to detect phosphorylated-IκBα (p-IκBα) and to determine the total amounts of IKKα and IKKβ in each sample. To determine whether α-tomatine directly targets IKK, IKKα and IKKβ immunoprecipitated from 10 ng/ml TNF-α and 0.1% DMSO (vehicle) treated cells. *In vitro* kinase assay was performed in the absence or presence of indicated concentrations of α-tomatine at 30°C for 30 minutes.

### Subcutaneous and orthotopic implantation of PC-3 cells

Male BALB/c nude mice (6 weeks old) were purchased from the Animal Resources Centre (Canning Vale, Western Australia). For subcutaneous tumor growth study, luciferase-expressing PC-3 prostate cancer cells (1×10^6^ in 0.1 ml Dulbecco’s PBS) were inoculated subcutaneously into the lower flanks of each mouse. On day 7 after cancer cell inoculation, each mouse had one palpable tumor and were randomly assigned to four groups (n  =  8/group). These groups of mice were then given intraperitoneal injections of vehicle solution, 10 mg/kg docetaxel, 5 mg/kg α-tomatine or 10 mg/kg α-tomatine thrice a week for an additional 3 weeks. All the mice were monitored weekly for tumor growth and body weight. Tumor dimensions were measured with calipers and volume calculated using a standard formula: (length×width^2^)×0.5 [25]. The experiment was terminated 28 days after cancer cell inoculation at which time bioluminescent signals of tumors in live mice were captured on a Xenogen IVIS Spectrum imaging system (Alameda, CA, USA) using Live Imaging Acquisition and Analysis software. Briefly, mice were injected intraperitoneally with luciferin potassium salt, anesthetized and luminescence images acquired. Luminescence signal intensity was quantified as region of interest analysis of total photons per second for each tumor. Tumors were excised, washed with ice-cold phosphate buffered saline (PBS) and stored at -80°C until examined by Western blot analysis.

Orthotopic growth of PC-3 cells was performed as described previously [19]. Briefly, under a dissecting microscope (Olympus, Tokyo, Japan), the prostate of 6 weeks old anesthetized SCID mice were exposed through a surgical incision and 2×10^5^ cells in 10 µl Dulbecco’s PBS injected into the dorsal prostate. Organs were then replaced, and the abdomen was closed in two layers with silk sutures. Five days after implantation, mice were randomly assigned to two groups that received vehicle solution or 10 mg/kg α-tomatine intraperitoneally thrice a week for 14 days (n  =  6 per group). Body weight was measured weekly. Bioluminescent signal of the PC-3 cell tumor in each mouse was measured at the end of the study using a Xenogen IVIS Spectrum imaging system as described above. All mice were then sacrificed by cervical dislocation.

### Tissue processing and protein extraction

For protein analysis of mouse tumors, tissues were minced, suspended in tissue protein extraction reagent (Thermo-Fisher Scientific, Waltham, MA) supplemented with complete protease and phosphatase inhibitors, and homogenized using a gentleMACS Dissociator (Miltenyi Biotec, Germany). The homogenized tissue was transferred to a pre-chilled microcentrifuge tube, incubated on ice for 30 minutes and then centrifuged at 15,000 x g for 20 minutes at 4°C. The supernatant containing total cellular proteins was collected for Western blot analysis. Nuclear proteins were extracted using a nuclear extraction kit according to the manufacturer’s instructions. Briefly, hypotonic buffer supplemented with 1 mM DTT and 0.01% Nonidet P-40 per gram of tissues was added to minced tissues, which were then homogenized with a gentleMACS Dissociator followed by incubation on ice for 15 minutes. Cytoplasmic proteins were separated by centrifugation. The pellets were resuspended in ice-cold complete nuclear extraction buffer then vortexed on ice for 30 seconds at highest setting. These cell pelleting and vortexing steps were repeated for a total of 6 cycles. The suspensions were then centrifuged at 14,000 x g for 10 minutes at 4°C and supernatants containing nuclear fractions were collected for Western blot analysis.

### Western blot analysis

Protein samples were separated by SDS-PAGE and then transferred onto PVDF membranes which were probed with primary antibodies, followed after washes by horseradish peroxidase-conjugated secondary antibodies and visualized colorimetrically after further washes using TMB solution. Protein bands were visualized and quantified using a gel documentation system (BioRad, Richmond, Calif).

### Statistical analysis

All assays were performed on at least three separate occasions. Results are expressed as the mean value ± standard error of the mean (SEM). Statistical analysis was performed with one-way analysis of variance, with Dunnett’s Multiple Comparison Test to identify between-group differences using GraphPad Prism software (version 5.0; GraphPad Software Inc., San Diego, CA). For *in vivo* tumor growth experiments, statistical significance of differences in tumor volume, body weight and total bioluminescence intensity between control and treatment groups was assessed by two-way ANOVA (GraphPad Software Inc., version 5.0, San Diego, CA), with p values < 0.05 considered significant. Statistical significance is expressed as ***, p<0.001; **, p<0.01; *, p<0.05.

## Results

### α-tomatine inhibits TNF-α-induced nuclear translocation of NF-κB p50 and p65 and phosphorylation of NF-κB p65

**Figure 1:**
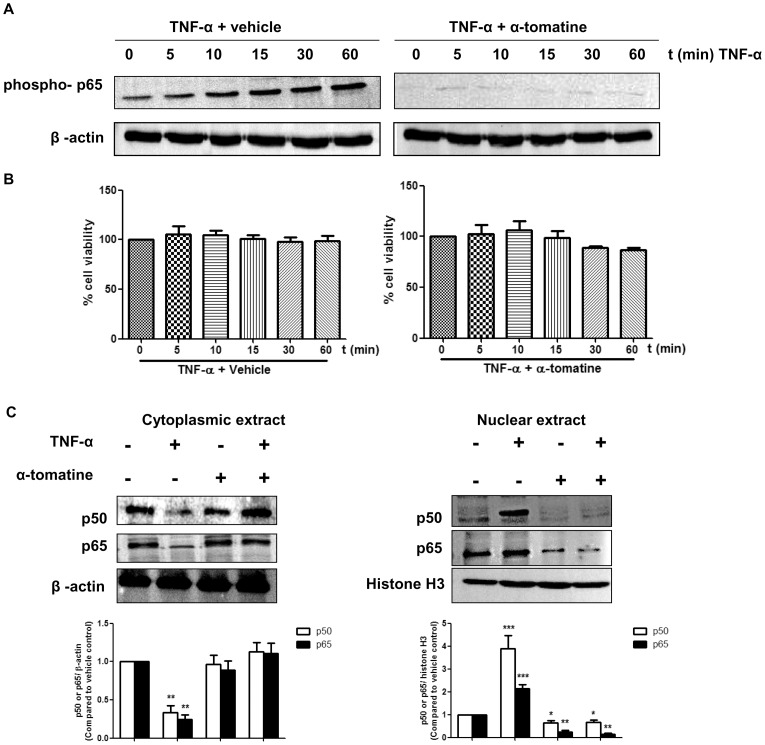
Effect of α-tomatine on TNF-α-induced phosphorylation of p65 and nuclear translocation of NF-κB p50/p65. (A) PC-3 cells at 70–80% confluency were treated with either 0.1% DMSO (vehicle) or 2 µM α-tomatine in DMSO for 30 minutes, followed by treatment with 10 ng/ml TNF-α for the indicated times. Cytoplasmic extracts were analyzed by Western blot analysis using an antibody against the phosphorylated form of p65. (B) Cell viability was assessed by counting cells that excluded trypan blue using a hemocytometer. (C) Effect of α-tomatine on nuclear and cytoplasmic levels of NF-κB p50 and p65 in human prostate cancer PC-3 cells. Nuclear and cytoplasmic fractions extracted from PC-3 cells treated either 0.1% DMSO or 2 µM α-tomatine in DMSO for 30 minutes, followed by treatment with 10 ng/ml TNF-α for the 30 minutes were analyzed by Western blot analysis with antibodies against NF-κB p50 and p65 proteins. β-actin and histone H3 proteins were loading control for cytoplasmic and nuclear extracts, respectively. Graphical representation of densitometry analysis of NF-κB p50 and p65 Western blot analyses from three independent experiments are shown below each panel. The ratio of the signal intensity of each protein to loading control was normalized to the vehicle control. * *P*<0.05, ** *P*<0.01, *** *P*<0.001 *vs* vehicle control.

We previously showed that α-tomatine inhibited the growth of androgen-independent human prostatic adenocarcinoma PC-3 *in vitro* with the half maximal effective concentration (EC_50_) value of 1.67 ± 0.3 μM [18]. At this chosen dose, α-tomatine was shown to be less cytotoxic to human normal prostate RWPE-1 (EC_50_ 3.85 ± 0.1 µM) and normal liver WRL-68 cells (EC_50_ > 5 µM) [18]. Treatment with 2 µM α-tomatine induces apoptosis and inhibits the TNF-α-induced NF-κB nuclear translocation on PC-3 cells [18]. In the present study, the mechanism of α-tomatine in inhibition of TNF-α-induced NF-κB nuclear translocation was investigated by analyzing its effect on phosphorylation and translocation of NF-κB sub-units in prostate cancer PC-3 cells. As shown in Figure 1A, the time-dependent phosphorylation of NF-κB p65 induced by TNF-α over a 60 minutes period was completely suppressed by pretreatment of cells with 2 µM α-tomatine. Examination of treated and control cell populations using a trypan blue exclusion assay indicated that the concentration of α-tomatine and TNF-α used and the time of exposure had minimal effect on cell viability (Figure 1B). In addition to the observed loss of p65 phosphorylation, α-tomatine prevented TNF-α induced translocation from the cytoplasm to the nucleus of both p50 and p65 NF-κB sub-units (Figure 1C). These findings indicate that α-tomatine inhibits the phosphorylation of NF-κB p65 and prevents the nuclear translocation of NF-κB p50 and p65.

### α-tomatine inhibits TNF-α-dependent IκBα phosphorylation and degradation

**Figure 2:**
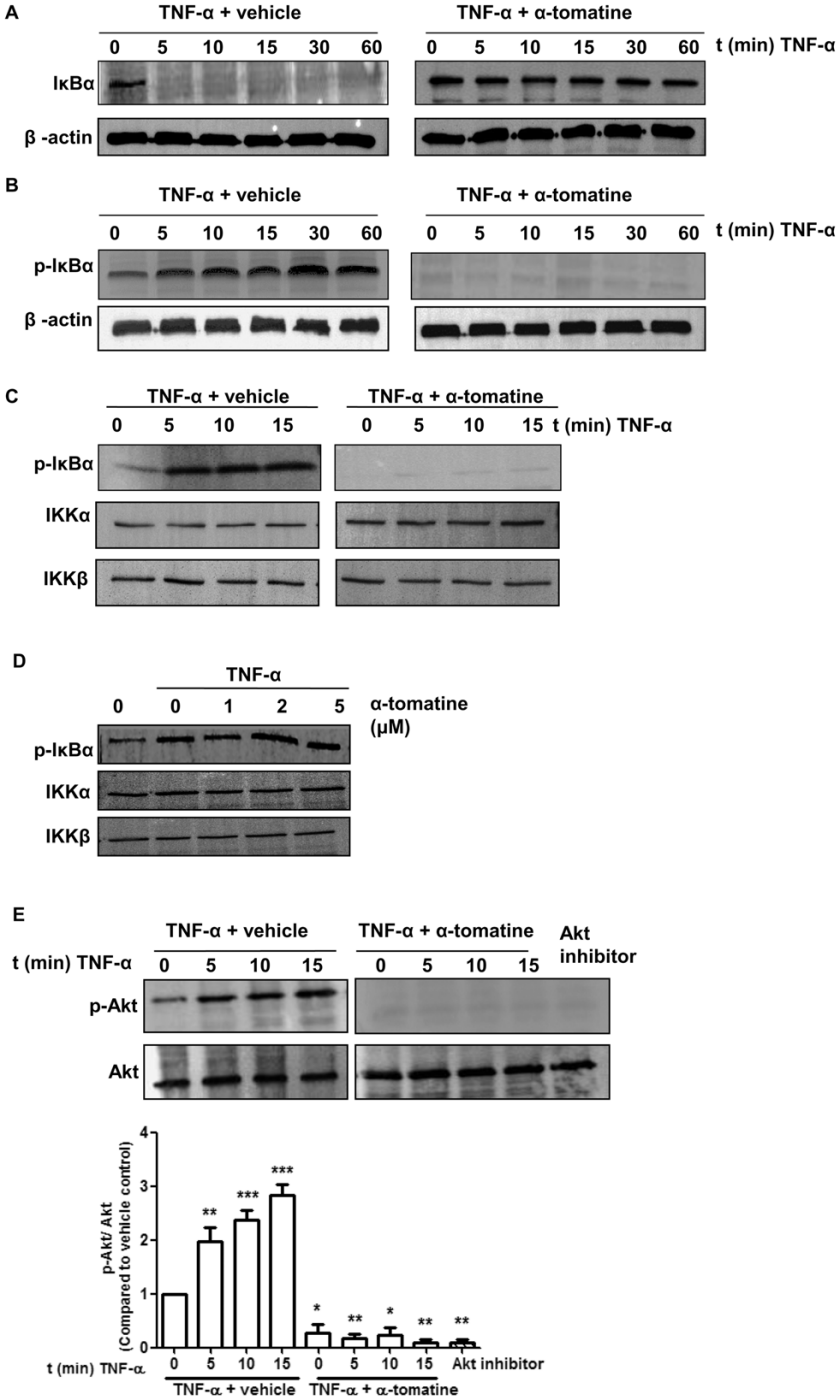
Effect of α-tomatine on IκBα Kinase activity. (A) Cells were grown to 70–80 % confluence, treated with either 0.1% DMSO (vehicle) or 2 µM α-tomatine in DMSO for 30 minutes, followed by treatment with 10 ng/ml of TNF-α for the indicated times. The presence of IκBα was detected by Western blot analysis. (B) To determine whether α-tomatine inhibits IκBα degradation by blocking IκBα phosphorylation, cells were treated with either 0.1% DMSO (vehicle) or 2 µM α-tomatine in DMSO for 30 minutes, followed by 50 µg/ml calpain inhibitor ALLN for 30 minutes, and then treated with 10 ng/ml of TNF-α for the times indicated. Anti-phospho-IκBα Western blot analysis was performed on cytoplasmic extracts. β-actin served as a loading control. (C) PC-3 cells were preincubated with either 2 µM α-tomatine or 0.1% DMSO (vehicle) for 30 minutes, and then treated with 10 ng/ml TNF-α for the indicated times. IKKα and IKKβ were immunoprecipitated from lysates from cells and *in vitro* kinase assays were performed using GST-IκBα as substrate as described in “Materials and Methods”. Western blot analysis was performed to detect phosphorylated IκBα. (D) IKK complex immunoprecipitated from vehicle and TNF-α-treated PC-3 cell extracts with an anti-IKKα and IKKβ antibodies was assayed for IKK activity. The kinase reaction mixture was incubated with α-tomatine as indicated. The expressions of phosphorylated IκBα and IKK were examined by Western blot analysis using anti-phospho-IκBα, anti-IKKα and anti-IKKβ antibodies. To examine the basal level of expression of IKK proteins, whole-cell extracts analyzed by Western blotting using anti-IKKα and anti-IKKβ antibodies. (E) PC-3 cells were pretreated with 2 µM α-tomatine for 30 minutes, and then treated with 10 ng/ml TNF-α for the indicated times. Lysates extracted from cells treated with 10 µM Akt inhibitor VIII for 3 hours serve as inhibition control. Cytoplasmic extracts were used for Western blotting using anti-phosphospecific Akt (Ser473) antibody. The same blot was reprobed with nonphosphorylated Akt antibody. Graphical representation of densitometry analysis of phosphor-Akt Western blot analysis from three independent experiments is shown below the panel. The ratio of the signal intensity of each protein to loading control was normalized to the vehicle control. * *P*<0.05, ** *P*<0.01, *** *P*<0.001 *vs* vehicle control.

The translocation of NF*-*κB to the nucleus is preceded by the phosphorylation, ubiquitination and proteolytic degradation of IκBα [26]. To determine whether the observed inhibition of TNF-α-induced NF*-*κB nuclear translocation caused by α-tomatine was due to inhibition of IκBα degradation, we pretreated cells with α-tomatine and then exposed them to TNF-α stimulation for time periods up to 60 minutes. TNF-α induced IκBα degradation in control cells as early as 5 minutes after stimulation (Figure 2A). In contrast, in α-tomatine-pretreated cells, TNF-α stimulation did not result in the degradation of IκBα, instead its expression was sustained up to 60 minutes of treatment (Figure 2B). The effect of α-tomatine on TNF-α-induced IκBα phosphorylation was examined using an antibody that detects IκBα phosphorylated at Ser32 and Ser36. Calpain inhibitor ALLN was used to prevent degradation of phosphorylated IκBα. Western blot analysis indicated that TNF-α induced IκBα phosphorylation as early as 5 minutes post-stimulation, but α-tomatine completely suppressed this event (Figure 2B). These results indicate that α-tomatine inhibited TNF-α-induced phosphorylation and degradation of IκBα and subsequent nuclear translocation of NF*-*κB.

### α-tomatine inhibits TNF-α-induced IKK activation

TNF-α induces IκBα phosphorylation and degradation via activation of the IKK complex [26]. To determine if pretreatment with α-tomatine affects IKK activation, *in vitro* kinase assays were performed using immunoprecipitated components of the IKK complex, and GST-IκBα as the IKK phosphorylation substrate. As shown in Figure 2C, TNF-α treatment stimulated IKK activity as phosphorylated-IκBα was detected as early as 5 minutes after treatment and remained detectable 10 minutes later. In contrast, phosphorylated-IκBα was not detected in cells treated with 2 µM α-tomatine during the 15 minutes period of TNF-α stimulation (Figure 2C). Western blot analysis demonstrated that TNF-α and α-tomatine had no effect on the expression of the components of the IKK complex, IKKα or IKKβ (Figure 2C), indicating that α-tomatine blocked TNF-α-induced phosphorylation of IκBα by attenuating the action of IKK rather than by causing degradation of this kinase. In a second set of experiments, we assessed whether α-tomatine suppressed IKK activity by directly binding to IKK protein by using IKKα and IKKβ immunoprecipitated from cells treated with TNF-α. The kinase reaction mixture was incubated with increasing concentrations of α-tomatine (1, 2 and 5 µM). As shown in Figure 2D, whereas TNF-α caused an increase in phosphorylation of IκBα, this was not reduced by inclusion of increasing concentrations of α-tomatine. This suggests that while α-tomatine efficiently inhibits IKK-mediated phosphorylation of IκBα, it did not do this by directly inhibiting IKK.

### α-tomatine inhibits TNF-α-induced Akt activation

It has been reported that the serine-threonine kinase Akt can activate IKK [27]. To gain insight into whether this pathway may be relevant in TNF-α–induced activation of NF-κB signaling, we pretreated PC-3 cells with 2 µM α-tomatine for 30 minutes then exposed these cells to TNF-α for time periods up to 15 minutes. In control experiments cells were treated with an Akt inhibitor, Akt inhibitor VIII, at 10 µM for 3 hours as described previously [22]. As shown in Figure 2E, TNF-α induced Akt activation in a time-dependent manner and α-tomatine suppressed this activation as effectively as Akt inhibitor VIII with no significant effect on the expression of total Akt protein.

### α-tomatine represses TNF-α-induced NF-κB dependent expression of pro-survival proteins

**Figure 3:**
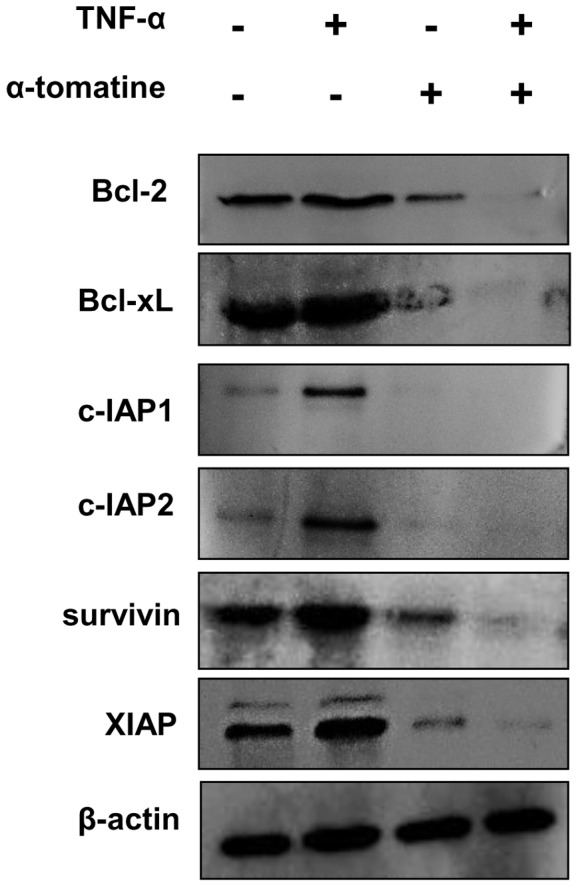
α-tomatine represses TNF-α-induced NF-κB dependent expression of pro-survival proteins. PC-3 cells grown to 70–80 % confluence were treated with either 0.1% DMSO or 2 µM α-tomatine for 30 minutes, 10 ng/ml TNF-α was then added and the cells were incubated for a further 6 hours. Whole-cell extracts were prepared, and were analyzed by Western blot analysis using antibodies against Bcl-2, Bcl-xL, c-IAP1, c-IAP2, XIAP and survivin. The results shown here are representative of three independent experiments.

Several studies have indicated that the transcription factor NF-κB regulates the expression of proteins implicated in facilitating tumor cell survival including Bcl-2, Bcl-xL, c-IAP, survivin and XIAP [28], [29]. Accordingly, we next examined whether α-tomatine inhibition of TNF-α-induced NF-κB nuclear translocation is accompanied by alterations in the expression of these pro-survival proteins. In these experiments cells were pretreated with α-tomatine (2 µM) for 30 minutes before induction of TNF-α-induced effects for 6 hours. This time period was selected to permit accumulation of levels of pro-survival proteins sufficient for Western blot analysis. As during this time period α-tomatine treatment resulted in varying levels of cell rounding and detachment in treated and control cells, both adherent and non-adherent cells were collected for protein extraction. As shown in Figure 3 Western blot analysis revealed that TNF-α induced marked upregulation of c-IAP1 and c-IAP2 while increases in expression of Bcl-xL, survivin and XIAP were also apparent but at lower levels and Bcl-2 expression was marginally increased in response to this cytokine. It was striking that α-tomatine caused sharp down-regulation of each of these mediators of cell survival under basal conditions as well as completely blocking TNF-α-induced upregulation of each protein (Figure 3). These data indicate that, consistent with its ability to inhibit TNF-α-induced NF-κB nuclear translocation, α-tomatine blocks the expression of the pro-survival mediators typically upreguated by this cytokine.

### α-tomatine attenuates growth of PC-3 cell xenograft tumors in mice

**Figure 4:**
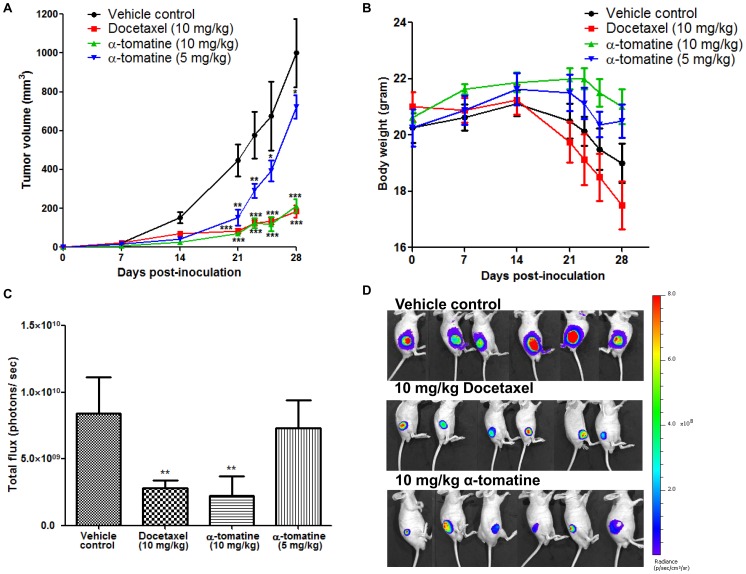
Anti-tumor activity of α-tomatine against subcutaneous PC-3 cell tumors. Luciferase expressing PC-3 cell xenograft tumors established in male nude mice (n  =  8 per treatment group) for 1 week were treated thrice weekly for 3 weeks with vehicle, docetaxel (10 mg/kg) or α-tomatine (5 or 10 mg/kg). (A) Graph of tumor volume in each treatment group versus the number of days after initial injection of PC-3 cells. (B) Graph of mean body weight for each treatment group versus the number of days after initial injection of PC-3 cells. (C) Bioluminescence intensities emitted from PC-3 cell xenograft tumors at the end of the experiment for each treatment group. (D) Bioluminescence images of PC-3 subcutaneous xenografts. The first row shows the vehicle control group; middle row shows the docetaxel treatment group; bottom row shows the 10 mg/kg α-tomatine treatment group. Each bar or point represents the mean ± SEM of data (n = 8).* *P*<0.05, ** *P*<0.01, *** *P*<0.001 *vs* vehicle control.

To examine the effect of α-tomatine on tumor growth *in vivo*, we challenged PC-3 cell tumors grown subcutaneously in mice. In these experiments tumors were allowed to establish for 1 week before challenge 3 times per week for 3 weeks with α-tomatine (5 or 10 mg/kg/mouse), the prostate cancer therapeutic docetaxel (10 mg/kg/mouse) [30] and vehicle solution. Figure 4A shows that 10 mg/kg α- tomatine was as efficient as 10 mg/kg docetaxel at retarding growth of PC-3 cell subcutaneous mouse tumors. In addition, there was also a marked decrease in tumor volume in response to lower dose of α-tomatine (5 mg/kg). Specifically, 3 weeks after the commencement of drug challenge the average tumor volume in vehicle control mice (1000 ± 180 mm^3^), was approximately 1.4 fold higher as compared to mice treated with 5 mg/kg α-tomatine (720 ± 60 mm^3^), and approximately 4.7 fold higher compared with mice treated with 10 mg/kg of α-tomatine (210 ± 35 mm^3^) or docetaxel (183 ± 32 mm^3^) (Figure 4A). Of note, α-tomatine treatment at both 5 mg/kg (Figure 4B, blue) and 10 mg/kg (Figure 4B, green) did not provoke body weight loss in contrast with docetaxel (Figure 4B, red) which caused a reduction in body weight of ∼10% more than those seen in untreated tumor bearing mice (Figure 4B, black). In fact, both control and docetaxel treatment groups showed a trend of decreasing body weight after 14 days of cancer cells inoculation, potentially due to high tumor burden and toxicity of docetaxel, respectively (Figure 4B). Consistent with the tumor volumes determined from caliper measurements, the intensity of bioluminescence measured on day 28 was significantly lower in both docetaxel and 10 mg/kg α-tomatine treatment groups (Figure 4C and D). These results suggest that α-tomatine retards the growth of PC-3 cell subcutaneous xenograft tumors at an effective dose of 10 mg/kg.

**Figure 5:**
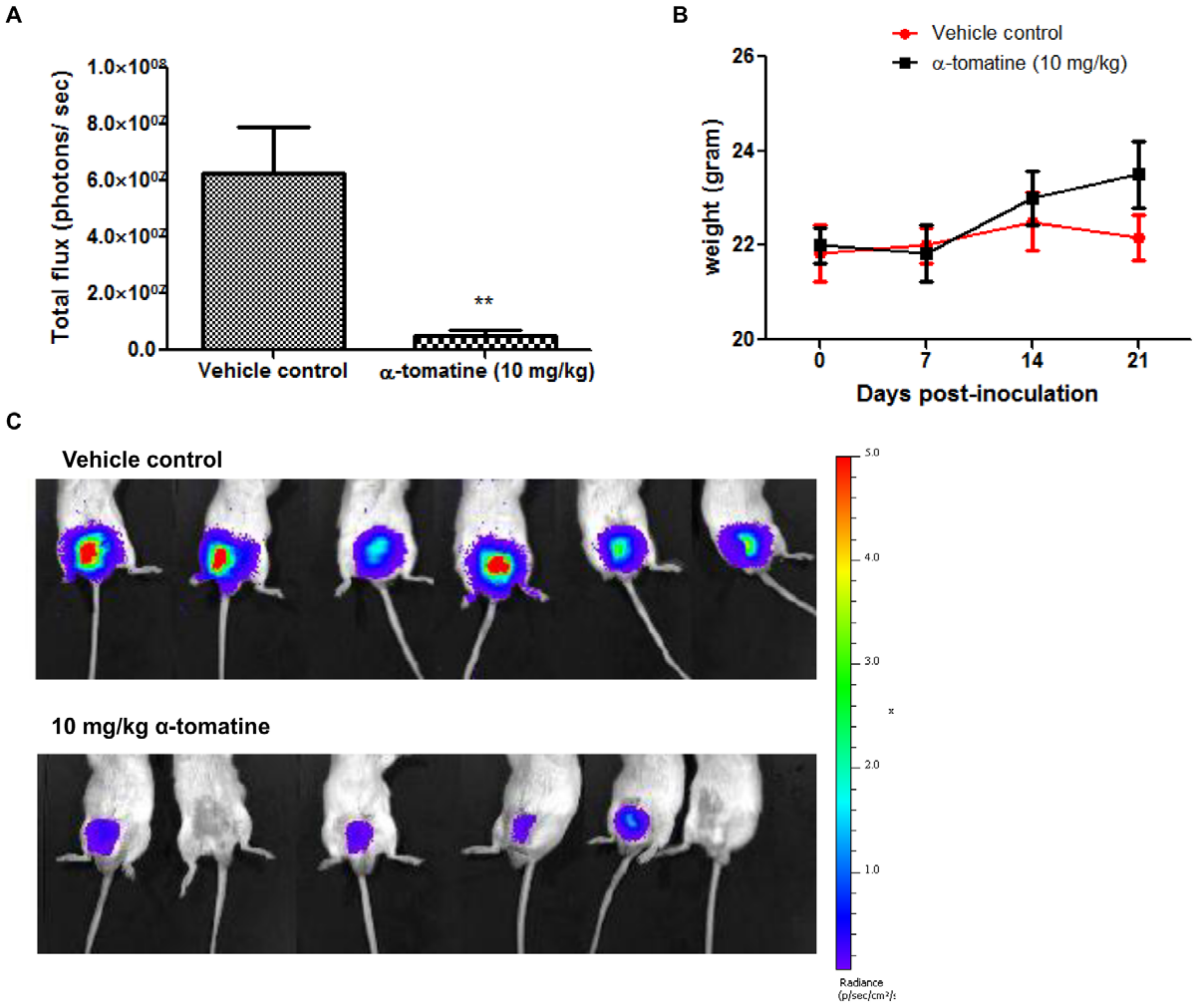
Anti-tumor activity of α-tomatine against orthotopic PC-3 cell tumors. Luciferase expressing PC-3 cell orthotopic tumors established in male SCID mice (n  =  6 per treatment group) for 5 days were treated thrice weekly for 2 weeks with vehicle or α-tomatine (10 mg/kg). (A) Bioluminescence intensities emitted from PC-3 cell orthotopic tumors for each treatment group after 14 days of treatment. (B) Bioluminescent images at the end of the experiment of SCID mice carrying orthotopic tumors of luciferase expressing PC-3-luc cells. The upper row shows the vehicle control group, whereas the bottom row shows the α-tomatine (10 mg/kg) treatment group. (C) Graph of mean body weight for each treatment group versus the number of days after initial injection of PC-3 cells. Each bar or point represents the mean ± SEM of data (n = 6).* *P*<0.05, ** *P*<0.01, *** *P*<0.001 *vs* vehicle control.

We also used an orthotopic mouse model to examine the effect of α-tomatine on prostate tumor growth. In this experiment, PC-3 cell tumors were allowed to grow in mice for 5 days before thrice weekly treatment for 2 weeks with 10 mg/kg α-tomatine. As shown in Figure 5A, and consistent with the data obtained from the xenograft mouse model, α-tomatine significantly suppressed the tumorigenicity of PC-3 cell orthotopic tumors. In addition, the total body weight of mice was not adversely affected by α-tomatine treatment (Figure 5B). The images in Figure 5C shows strong bioluminescent signal from the prostate of control mice, demonstrating that the tumor cells were successfully implanted. In addition, 4 out of 6 α-tomatine treated mice showed only weak bioluminescent signals and another 2 mice had no signal. These findings demonstrate that α-tomatine has potent anti-tumor effects against mouse xenograft and orthotopic PC-3 cell tumors.

### α-tomatine reduces expression of proliferation markers, increases expression of apoptosis markers and inhibits nuclear translocation of NF- κB in xenograft tumors

**Figure 6:**
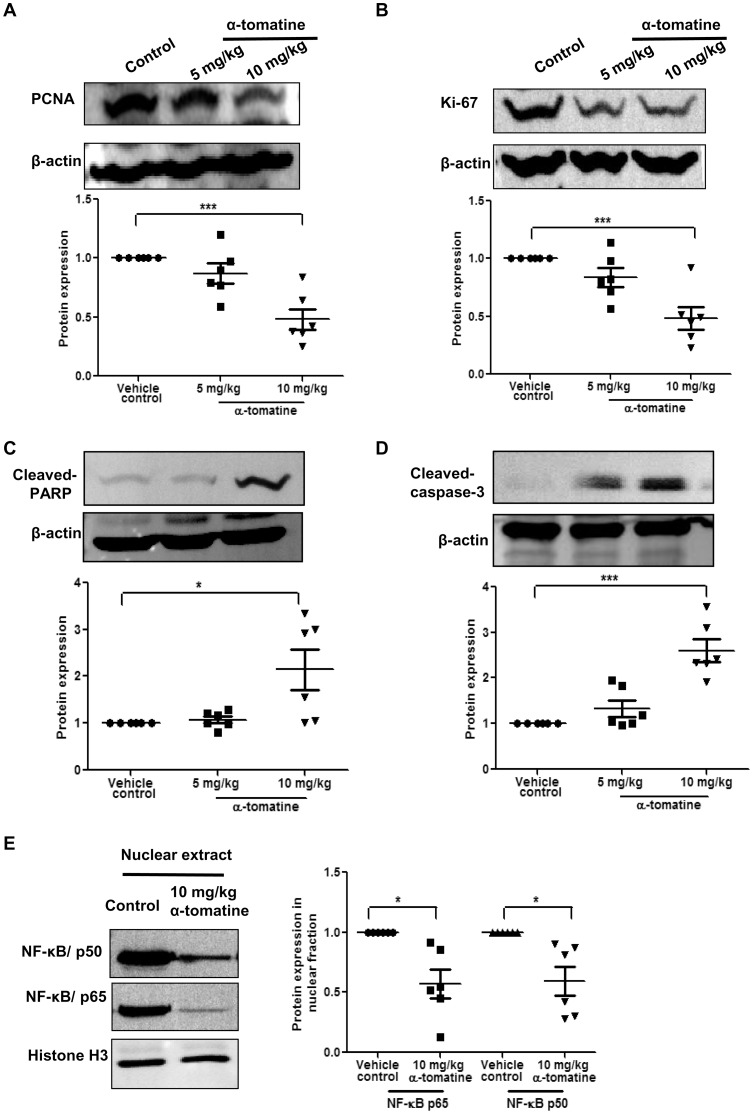
Western blot analysis of PCNA, Ki-67, cleaved-PARP, cleaved-caspase-3 and NF-κB in PC-3 tumor tissues samples. (A–D) PC-3 tumors excised from mice treated with vehicle, 5 mg/kg or 10 mg/kg of α-tomatine were lysed and examined by Western blot analysis. Representative images are shown. Markers of proliferation (PCNA and Ki-67). Markers of apoptosis (cleaved PARP and cleaved caspase-3). β-actin was used as loading control. (E) Western blot analysis on nuclear extracts probing for NF-κB p50 and p65 levels. Equal loading of protein was confirmed by stripping and reprobing the blots with histone H3 antibody. Graphical representation of densitometry analysis of each Western blot data from six independent tumor samples is shown below each panel. The ratio of the signal intensity of each protein to loading control was normalized to the vehicle control. * *P*<0.05, ** *P*<0.01, *** *P*<0.001 *vs* vehicle control.

To examine the mechanism by which α-tomatine suppressed the growth of PC-3 cell tumors in mice, we next examined tumor tissue recovered from mice carrying subcutaneous tumors for expression of markers of proliferation (PCNA and Ki-67) and apoptosis (cleaved-PARP and cleaved-caspase-3). This analysis was not possible for orthotopic tumors as insufficient material was available for the α-tomatine treated mice. As shown in Figure 6, Western blot analysis of lysates from six independent tumor samples indicated that while both markers of proliferation (PCNA and Ki-67) decreased in response to α-tomatine treatment (Figure 6A and B), levels of both markers of apoptosis (cleaved-PARP and caspase-3) increased (Figure 6C and D). These data suggest that both anti-proliferative and pro-apoptotic effects of α-tomatine contribute to the reduced growth of PC-3 cell tumors in mice. As NF-κB translocation to the nucleus is important for promoting increased cell proliferation and survival and we have shown that this translocation event is reduced *in vitro* by α-tomatine, we were interested to examine recovered subcutaneous mouse tumors for localization of NF-κB. As shown in Figure 6E, Western blot analysis from six independent tumor samples indicated that there was a distinct decrease in the levels of NF-κB components in the nucleus of tumor cells in response to α-tomatine treatment. These data suggest that the anti-tumor effects of α-tomatine *in vivo* may be due to its ability to block the proliferative and anti-apoptotic effects of NF-κB signaling by reducing its translocation to the nucleus.

## Discussion

We provide the first evidence that the saponin α-tomatine can efficiently inhibit the growth of prostate cancer tumors *in vivo* without inducing overt toxicity. Our analysis of recovered mouse tumors suggest that mechanistically α-tomatine mediates its anti-prostate cancer effects *in vivo* by blocking, at least in part, the proliferative and anti-apoptotic effects of NF-κB signaling by reducing translocation of this protein complex to the nucleus. Our detailed *in vitro* analyses indicate that α-tomatine suppression of NF-κB activation occurs through indirect inhibition of IKK kinase activity, leading to suppression of IκBα phosphorylation and degradation, NF-κB/p65 phosphorylation and NF-κB p50/p65 nuclear translocation. Consistent with its ability to induce apoptosis, α-tomatine inhibition of NF-κB activation *in vitro* was accompanied by significant reduction in expression of TNF-α induced pro-survival mediators c-IAP, Bcl-2, Bcl-xL, survivin and XIAP.

The NF-κB signaling pathway is an important target for disease treatment because its dysregulation is required for inappropriate inflammatory responses as well as cancer and other ailments [31]. In response to most activating stimuli, NF-κB signaling occurs through sequential activation of IKK, phosphorylation of IκBα at serine 32 and 36, leading to its degradation, and the nuclear translocation of NF-κB where it regulates transcription of a range of genes including those that promote cell proliferation and survival [32]. Consistent with our data from α-tomatine, suppression of NF-κB activation has been shown to be a critical mechanism of action of several plant-derived anticancer agents, such as curcumin, lycopene, silibinin, genistein, resveratrol and green tea polyphenols [33]. In the present study, we showed that α-tomatine is an indirect inhibitor of IKK. Presumably, α-tomatine inhibits upstream signaling components that lead to activation of the IKK complex, such as Akt serine-threonine kinase , NF-κB inducing kinase, mitogen-activated protein kinase kinase kinase (MEKK)1, MEKK3, TGF activated kinase 1 (TAK1) and glycogen synthase kinase-3 beta [34], [35], [36], [37], [38]. Indeed, we found that α-tomatine inhibits TNF-α-induced Akt activation, although further experiments are needed to address if this effect is responsible for the changes we observed in phosphorylation and translocation of NF-κB components or the anti-tumor actions of α-tomatine against subcutaneous and orthotopic tumors grown in mice. These results are in agreement with those of previous studies that suggest that α-tomatine suppresses invasion and migration of human lung cancer cells *in vitro* through the inhibition of the Akt [15], [16]. As conventional chemotherapeutic agents, including docetaxel target normal as well as tumor cells and lead to deleterious effects for prostate cancer patients there is a pressing need to identify less toxic agents to control this disease. In recent years, a number of naturally occurring dietary agents of reduced toxicity have been reported to induce apoptosis and inhibit tumor growth, highlighting the promise of using naturally derived agents for chemotherapy and chemoprevention of prostate and other cancers [39], [40], [41].

Furthermore, it is also possible that an inhibitor of NF-κB activation could be an adjuvant for overcoming tumor resistance to radiation and chemotherapies, such as paclitaxel, doxorubicin, 5-fluorouracil, and vinca alkaloids (vinblastine and vincristine), that occur via NF-κB activation [42], [43], [44]. It is thought that this induced resistance in a wide variety of tumor cells occurs via induction of NF-κB effector genes, including Bcl-2 [45], Bcl-xL [46], survivin [47], XIAP [48], c-IAP1 [49] and c-IAP2 [49] that are known to mediate protective responses to chemotherapeutic agents and radiation. Therefore, targeting NF-κB through the actions of α-tomatine, which we have shown to block NF-κB activation and transcription of NF-κB effector genes, may result in improvements in treatment of prostate cancer. In support of this, other dietary agents, including resveratrol, curcumin, genistein, (−) epigallocatechin gallate and soya isoflavone that can block various steps leading to NF-κB activation and sensitize tumor cells to the beneficial effects of chemotherapeutic drugs and radiation in treatment of cancer [50], [51], [52], [53], [54], [55], [56].

We previously reported that α-tomatine induces caspase-dependent death of PC-3 cells *in vitro* accompanied by increased caspase-3 activity and the release of cytochrome c [18]. Caspases have been shown to be involved in apoptosis through activation of PARP downstream molecule [57]. Importantly, here we have demonstrated the pro-apoptotic effect of α-tomatine *in vivo* by showing a significant increase in the cleavage of PARP and caspase-3 in α-tomatine-treated tumors. In addition, our data show that α-tomatine elicits anti-proliferative effects *in vivo* as we observed reduced levels of the markers of proliferation Ki-67 and PCNA. Our data from PC-3 tumors also suggest that these pro-apoptotic and anti-proliferative effects of α-tomatine are mediated, at least in part, by reduced nuclear translocation of NF-κB p50 and p65. Indeed our *in vitro* studies clearly demonstrate that α-tomatine is very effective at blocking activation and translocation of the components of the NF-κB complex providing support for the possibility that this mechanism is also important for its effects *in vivo* against prostate cancer PC-3 cell tumors.

In summary, we present the first evidence that α-tomatine is an effective anti-tumor compound against prostate cancer xenograft and orthotopic tumors. This agent may prove to be useful in the prevention and treatment of androgen-independent prostate cancer and this warrants further investigation.
