# Correction: Alpha-Tomatine Attenuation of *In Vivo* Growth of Subcutaneous and Orthotopic Xenograft Tumors of Human Prostate Carcinoma PC-3 Cells Is Accompanied by Inactivation of Nuclear Factor-Kappa B Signaling

**DOI:** 10.1371/journal.pone.0268234

**Published:** 2022-05-03

**Authors:** Sui-Ting Lee, Pooi-Fong Wong, Hui He, John David Hooper, Mohd Rais Mustafa

During the preparation of Fig 2 the incorrect IKKβ data were used inadvertently, so that the Fig 2C TNF-α + tomatine IKKβ panel and the Fig 2D IKKβ panel are incorrect. The correct panels have been included in the updated version of Fig 2 provided with this notice.

**Fig 2:**
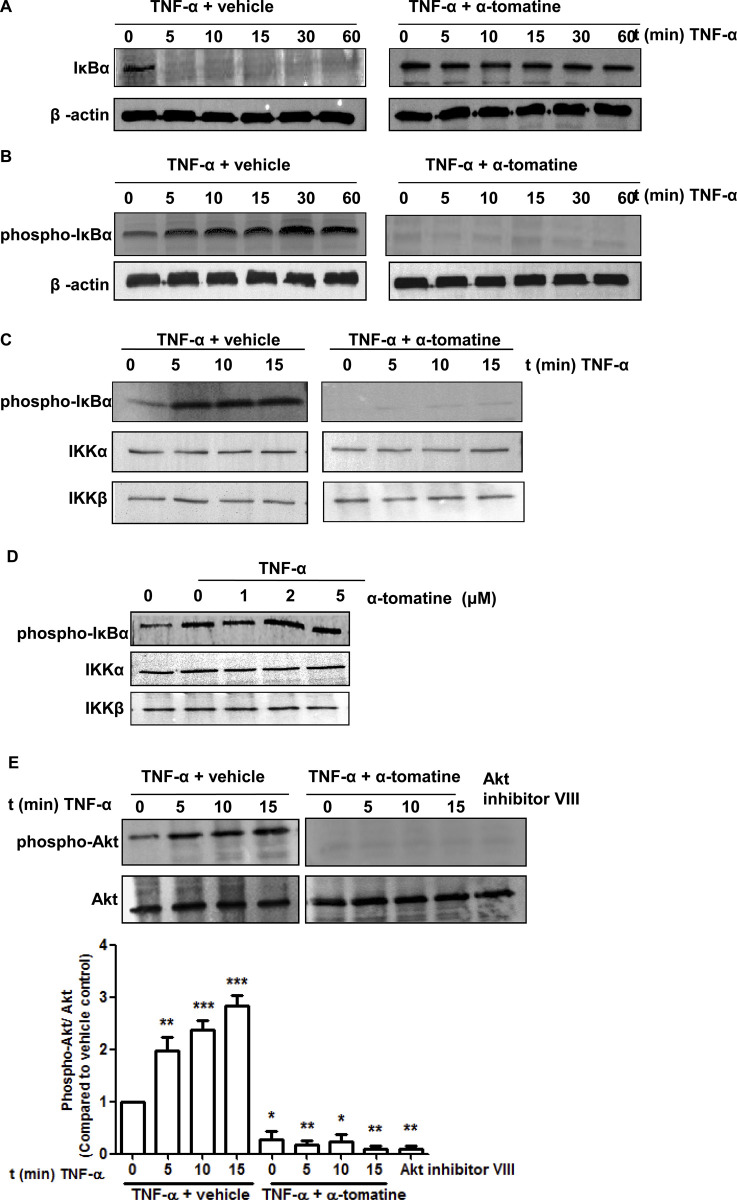
Effect of α-tomatine on IκBα Kinase activity. (A) Cells were grown to 70–80% confluence, treated with either 0.1% DMSO (vehicle) or 2 µM α-tomatine in DMSO for 30 minutes, followed by treatment with 10 ng/ml of TNF-α for the indicated times. The presence of IκBα was detected by Western blot analysis. (B) To determine whether α-tomatine inhibits IκBα degradation by blocking IκBα phosphorylation, cells were treated with either 0.1% DMSO (vehicle) or 2 µM α-tomatine in DMSO for 30 minutes, followed by 50 µg/ml calpain inhibitor ALLN for 30 minutes, and then treated with 10 ng/ml of TNF-α for the times indicated. Anti-phospho-IκBα Western blot analysis was performed on cytoplasmic extracts. β-actin served as a loading control. (C) PC-3 cells were preincubated with either 2 µM α-tomatine or 0.1% DMSO (vehicle) for 30 minutes, and then treated with 10 ng/ml TNF-α for the indicated times. IKKα and IKKβ were immunoprecipitated from lysates from cells and *in vitro* kinase assays were performed using GST-IκBα as substrate as described in “Materials and Methods”. Western blot analysis was performed to detect phosphorylated IκBα. (D) IKK complex immunoprecipitated from vehicle and TNF-α-treated PC-3 cell extracts with an anti-IKKα and IKKβ antibodies was assayed for IKK activity. The kinase reaction mixture was incubated with α-tomatine as indicated. The expressions of phosphorylated IκBα and IKK were examined by Western blot analysis using anti-phospho-IκBα, anti-IKKα and anti-IKKβ antibodies. To examine the basal level of expression of IKK proteins, whole-cell extracts analyzed by Western blotting using anti-IKKα and anti-IKKβ antibodies. (E) PC-3 cells were pretreated with 2 µM α-tomatine for 30 minutes, and then treated with 10 ng/ml TNF-α for the indicated times. Lysates extracted from cells treated with 10 µM Akt inhibitor VIII for 3 hours serve as inhibition control. Cytoplasmic extracts were used for Western blotting using anti-phosphospecific Akt (Ser473) antibody. The same blot was reprobed with nonphosphorylated Akt antibody. Graphical representation of densitometry analysis of phosphor-Akt Western blot analysis from three independent experiments is shown below the panel. The ratio of the signal intensity of each protein to loading control was normalized to the vehicle control. * *P*<0.05, ** *P*<0.01, *** *P*<0.001 vs vehicle control.

The label of the color scale in Fig 5C has been cropped during figure preparation. The figure has been corrected to include the uncropped color scale in the updated version of Fig 5C provided here. The original data underlying Fig 5 are no longer available.

**Fig 5:**
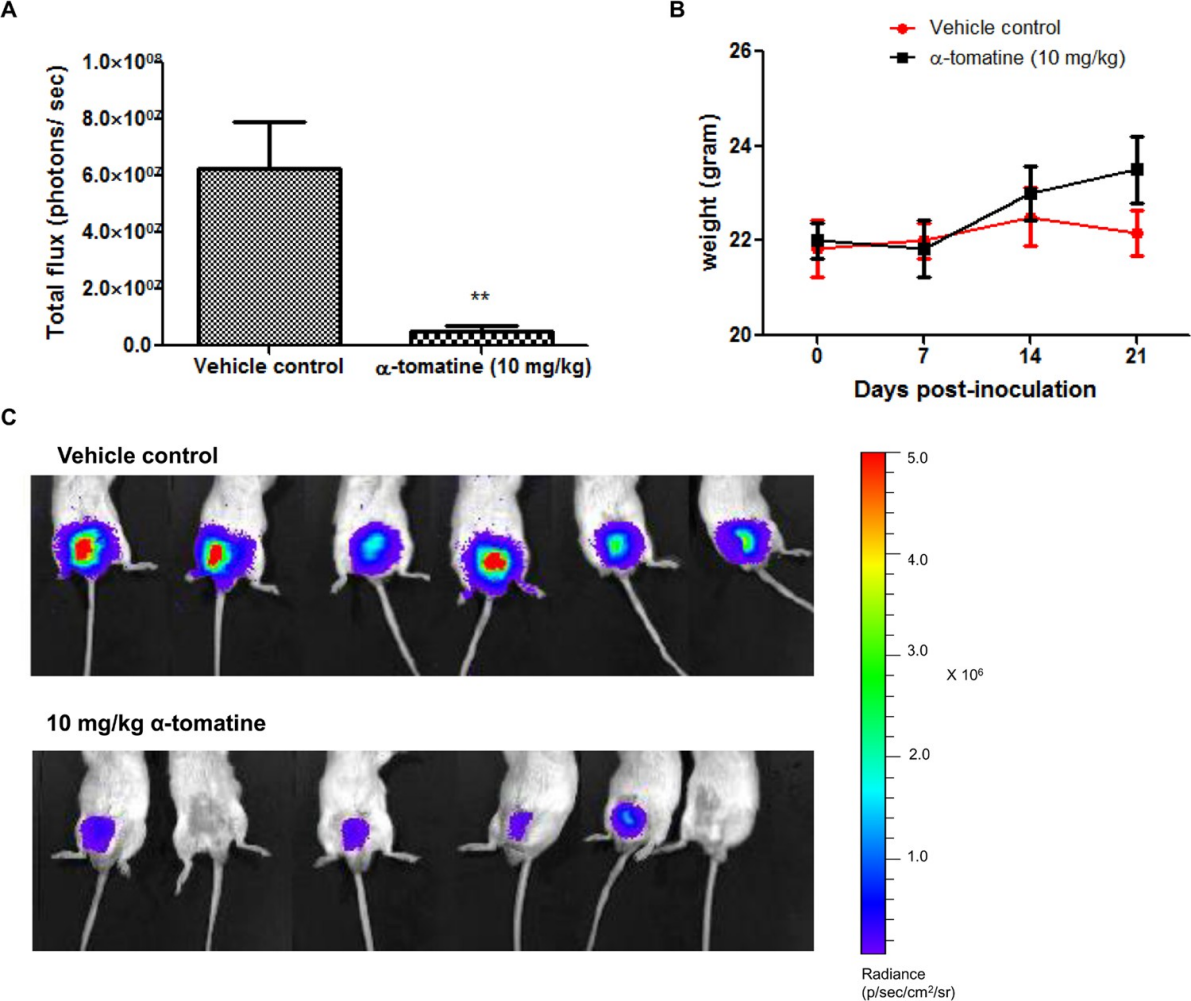
Anti-tumor activity of α-tomatine against orthotopic PC-3 cell tumors. Luciferase expressing PC-3 cell orthotopic tumors established in male SCID mice (n =  6 per treatment group) for 5 days were treated thrice weekly for 2 weeks with vehicle or α-tomatine (10 mg/kg). (A) Bioluminescence intensities emitted from PC-3 cell orthotopic tumors for each treatment group after 14 days of treatment. (B) Bioluminescent images at the end of the experiment of SCID mice carrying orthotopic tumors of luciferase expressing PC-3-luc cells. The upper row shows the vehicle control group, whereas the bottom row shows the α-tomatine (10 mg/kg) treatment group. (C) Graph of mean body weight for each treatment group versus the number of days after initial injection of PC-3 cells. Each bar or point represents the mean ± SEM of data (n  =  6).* *P*<0.05, ** *P*<0.01, *** *P*<0.001 vs vehicle control.

The available data underlying the Fig 2C and 2D panels are provided in the S1 File below. The original data underlying Fig 2A, 2B and 2E, and Fig 5 are no longer available due to the time elapsed since the experiments took place.

The authors provided updated [1] versions of Figs 2 and 5 in a public comment posted on this article in 2014, and they submitted the underlying data presented in the S1 File and updated figures for this article for editorial review by PLOS in 2015. The *PLOS ONE* Editors sincerely regret that this case was not resolved much sooner after the prior correspondence.

## Supporting information

S1 File Available data underlying Fig 2C and 2D. (PPT)
