# Supplementary figures and images for: Correction: Alpha-Tomatine Attenuation of In Vivo Growth of Subcutaneous and Orthotopic Xenograft Tumors of Human Prostate Carcinoma PC-3 Cells Is Accompanied by Inactivation of Nuclear Factor-Kappa B Signaling
Source: PLoS One. 2022 May 3;17(5):e0268234. doi: 10.1371/journal.pone.0268234 (PMC9064074; doi:10.1371/journal.pone.0268234)

## Slide 1
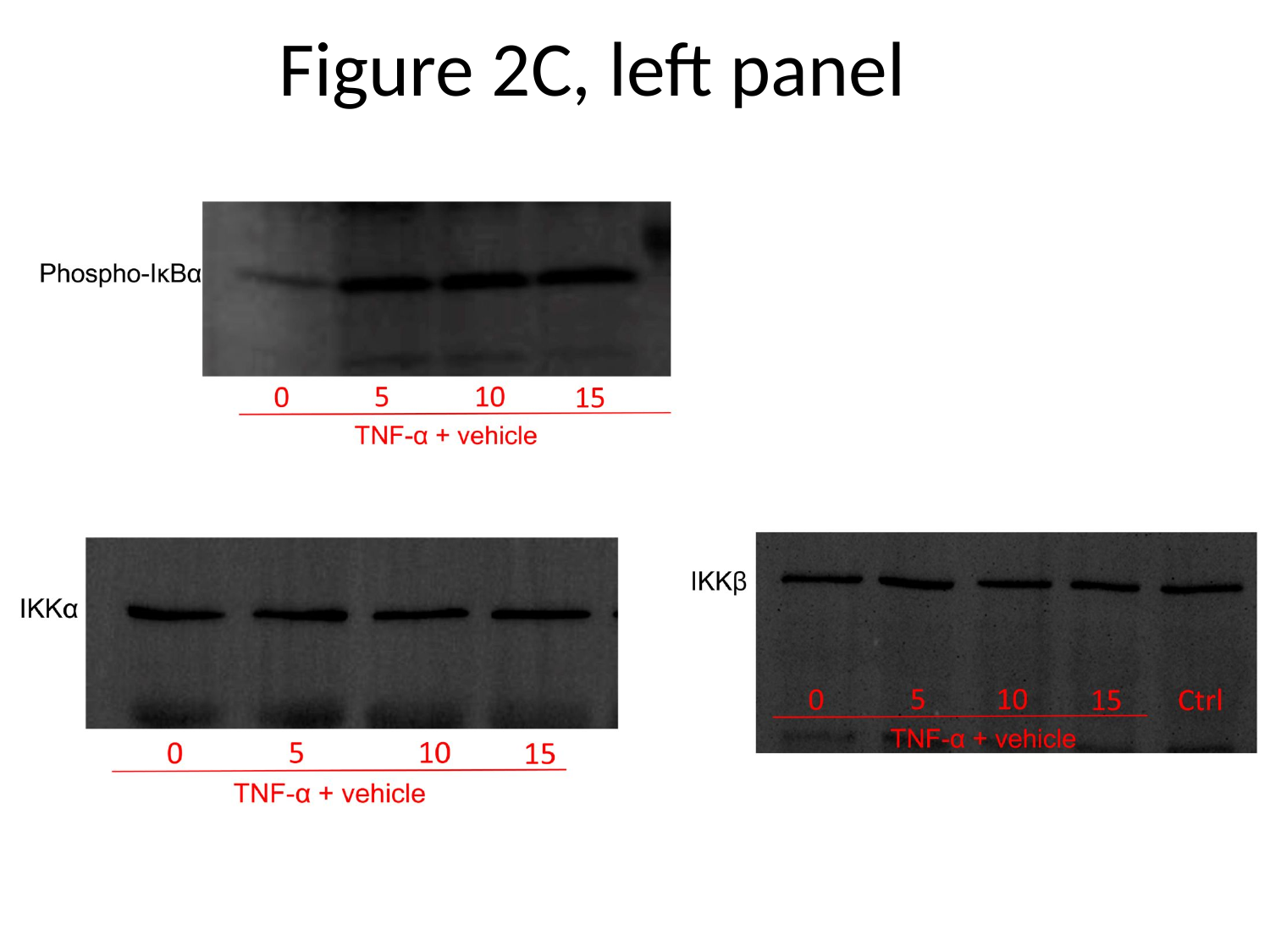

# Figure 2C, left panel

## Slide 2
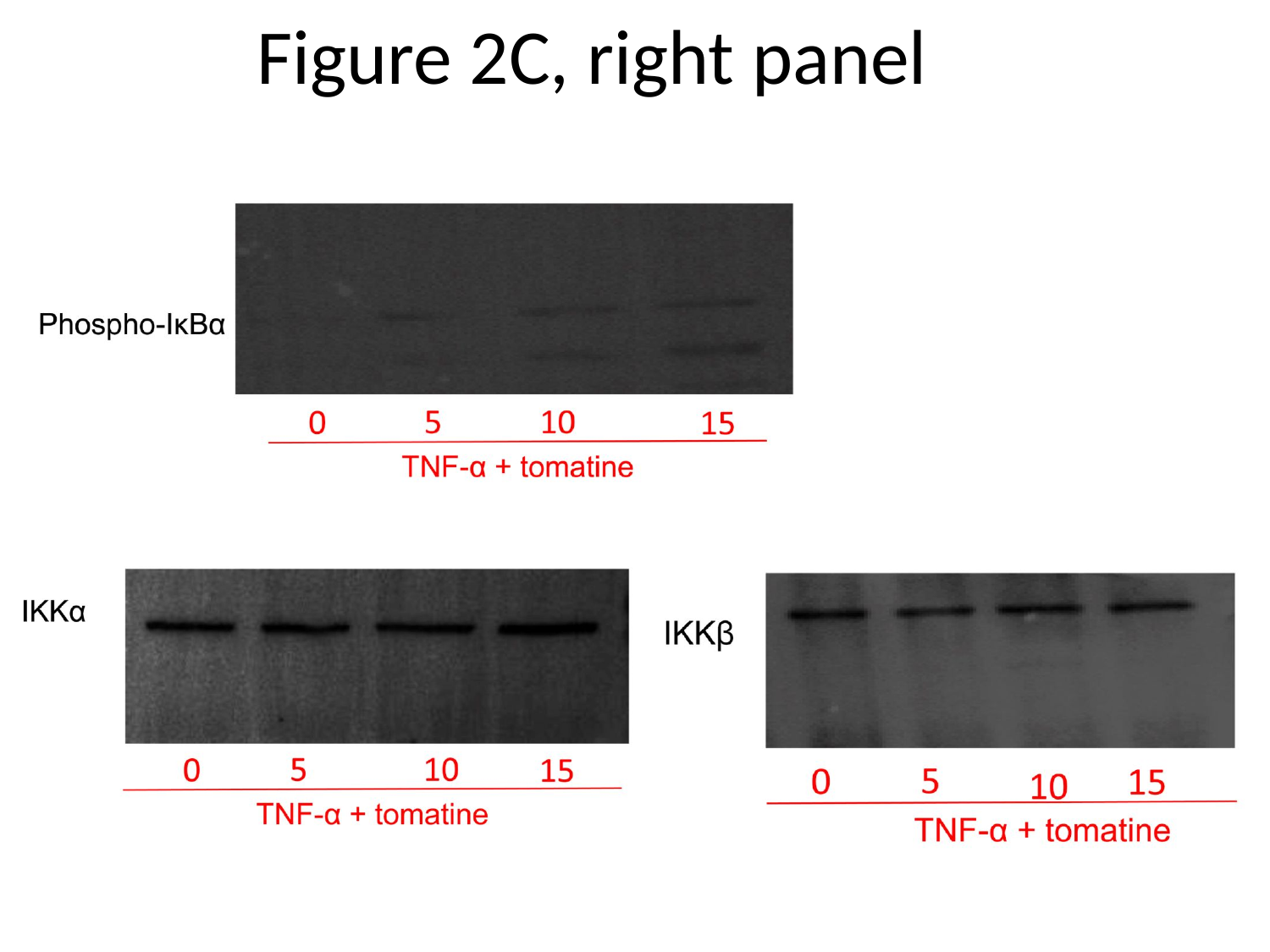

Figure 2C, right panel

## Slide 3
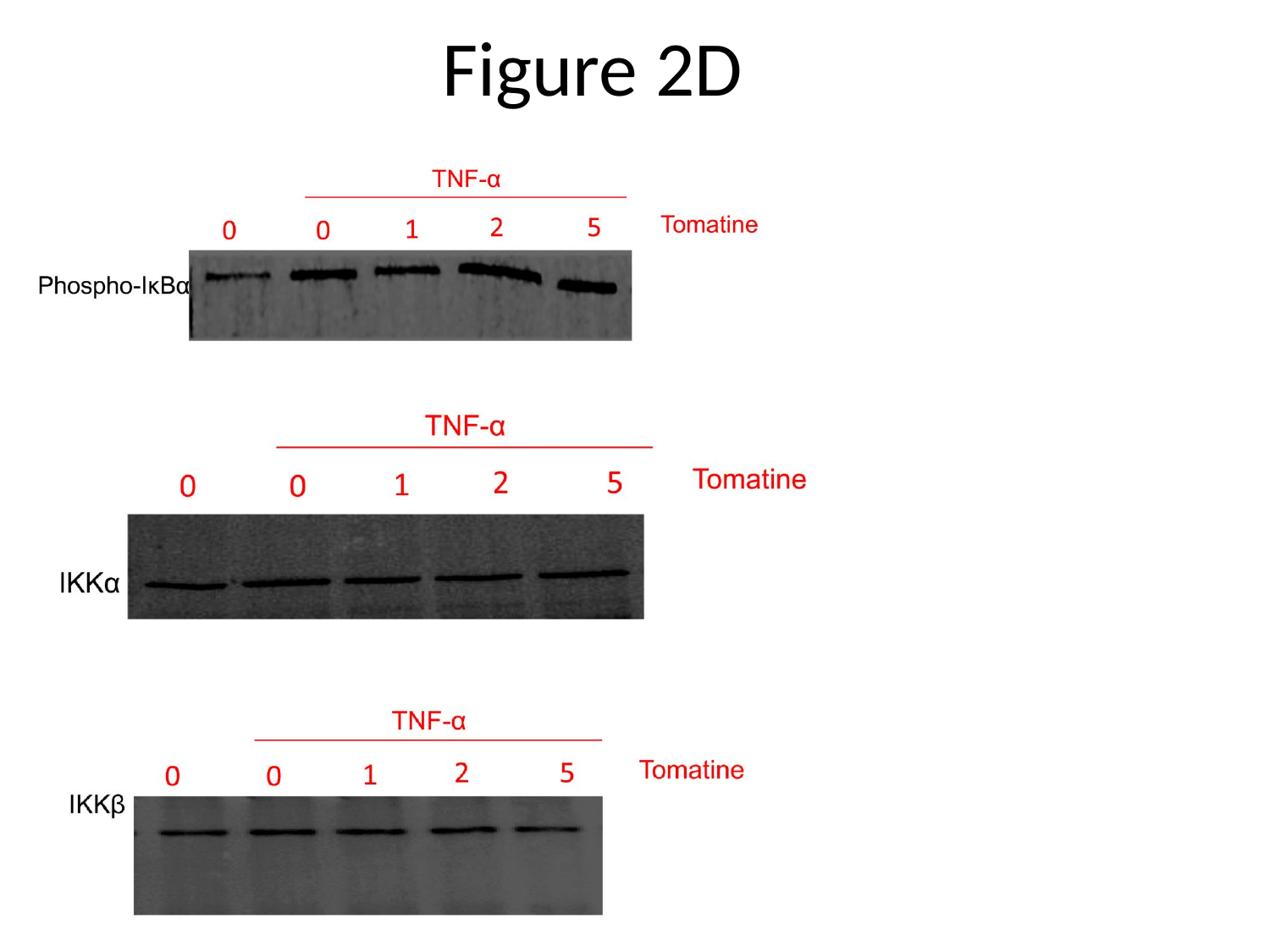

Figure 2D

Supplement: S1 File — (PPT) [file pone.0268234.s001.ppt]
